# COVID-19 and its treatments: lights and shadows on testicular function

**DOI:** 10.1007/s12020-022-03221-6

**Published:** 2022-10-19

**Authors:** Francesco Pallotti, Sandro C. Esteves, Fabiana Faja, Alessandra Buonacquisto, Anna Chiara Conflitti, Maria Neve Hirsch, Andrea Lenzi, Donatella Paoli, Francesco Lombardo

**Affiliations:** 1 Laboratory of Seminology – Sperm Bank “Loredana Gandini”, Department of Experimental Medicine, Sapienza Università di Roma, Rome, Italy; 2 Andrology and Human Reproduction Clinic, Av. Dr. Heitor Penteado, 1464 Campinas, Brazil; 3 Faculty of Health, Department of Clinical Medicine, Aarhus University, Aarhus, Denmark

**Keywords:** SARS-CoV-2, COVID-19, Andrological health, Male fertility, Spermatogenesis, Cytokine

## Abstract

**Purpose:**

The SARS-CoV-2 pandemic has rapidly spread worldwide and, among the others, the male gender was quickly recognized as an independent risk factor for both the disease and its consequences. Since the possibility of long-term hormonal axis changes and male gamete impairment have been hypothesized but a relatively low levels of evidence has been reached, we focused this narrative mini-review on summarizing key state-of-the-art knowledge on male reproductive effects of COVID-19 as a quick reference for reproductive health specialists.

**Methods:**

A comprehensive Medline/PubMed and Embase search was performed selecting all relevant, peer-reviewed papers in English published from 2020. Other relevant papers were selected from the reference lists.

**Results:**

Available evidence indicates that the likelihood of direct testicular damage from SARS-CoV-2 is somewhat low, but there are many indirect ways (fever, cytokine imbalance, and drugs) through which the pituitary-gonadal axis and spermatogenesis may be disrupted. These alterations are probably transient, but as available evidence is low quality, it cannot be excluded that previous pathologies or comorbidities might modulate the risk of their persistence. On the other hand, available evidence shows high safety regarding andrological health for available vaccines, although studies are mainly focused on mRNA vaccines.

**Conclusion:**

A careful andrological evaluation of men recovering from COVID-19 is highly recommended. Since available evidence is relatively scarce, a careful andrological follow-up and counseling of these patients are mandatory.

## Introduction

Since 2020, the SARS-CoV-2 pandemic has rapidly spread and caused millions of deaths worldwide [1]. The male gender, in particular, was quickly recognized as an independent risk factor for both the disease and its consequences [2]. This has been attributed to the contribution of multiple factors, possibly a combination of sex-specific hormone balance, genetic background, and behavioral/lifestyle patterns [3]. Therefore, at first researchers focused on key reproductive health issues, that is whether SARS-CoV-2 presence could be detected in seminal fluid and any possible direct influence on testicular function [4]. Following this first phase, vaccination campaigns and acquired immunity have allowed the industrialized countries to contain the emergency, rapidly shifting the attention of clinicians and researchers from the immediate consequences of the infections to its mid- and long-term effects. The persistence of symptoms in the post-acute phase has been reported in many subjects, and possible neurological and cardiovascular consequences have been described [5]. Thus, the interest of reproductive health researchers concentrated on possible alterations of semen parameters, reproductive outcomes, and other aspects of patient care [3, 4, 6]. Finally, fear of consequences and their possible negative impact on the healthcare systems of the countries has driven many researchers to divert resources to the study of the so-called post-COVID syndrome in terms of cardiovascular, pneumological, and neurological health [7], which, however, still needs to find full consensus. In this perspective, many have postulated the chance of long-term reproductive effects of SARS-CoV-2 in terms of both hormonal axis changes and gamete impairment [8, 9]. Unfortunately, as SARS-CoV-2 might be capable of causing damage to reproductive health indirectly through a variety of mechanisms, including side effects of its treatments [3], reproductive health specialists require to focus on several peculiar pathophysiological aspects in order to appropriately evaluate the post-COVID patient. Due to the large amount of data published but relatively low levels of evidence reached, we focused this mini-review on summarizing the state-of-the-art knowledge on reproductive effects of COVID-19, in order to condensate in an easy-to-read text the most relevant issues for a reproductive health endocrinologist.

## Material and methods

Relevant papers for this narrative review were retrieved from a comprehensive Medline/PubMed and Embase search. The database search was performed using the following keywords “COVID-19”, “SARS-CoV-2”, “testes”, “spermatogenesis”, “spermatozoa”, “testosterone”, “male infertility”, “glucocorticoids”, “semen quality”, “Tocilizumab”, “remdesivir”, “SARS-CoV-2 management”. Relevant, peer-reviewed papers in English published from 2020 have been considered. Other relevant papers were selected from the reference lists.

### Gender and COVID-19: the roots of the threat to male testicular function

Very early in the short history of the SARS-CoV-2 pandemic, it was clear that the male gender was a risk factor for severe disease and an increased mortality rate [10, 11]. Multiple reasons could justify this finding, but realistically the causality of this association can be found in a mixture of lifestyle patterns and other intrinsic gender-related biological characteristics, including comorbidities, genetic differences, and hormonal profile [2]. The angiotensin-converting enzyme 2 (ACE2) is exploited by SARS-CoV-2 as its receptor, through which it can invade the host cells. ACE2 is highly expressed in human tissues, including Leydig and Sertoli cells, seminiferous duct cells, and spermatogonia, allowing to postulate their potential role as a viral target [12]. Nonetheless, SARS-CoV-2 also requires additional mediators to facilitate the virus-host cell fusion [13]; transmembrane protease serine 2 (TMPRSS2), in particular, by cleaving the spike protein, might have a role in the male reproductive system as its expression is regulated by androgens and it is known to be expressed in human prostate epithelial cells [14]. Although evidence of simultaneous expression of these two proteins in the male genital trait is limited [15, 16], this theoretical background has been generally accepted to hypothesize the risk of testicular infection and, thus, of testicular COVID-related damage, further supported by post-mortem detection of viral proteins in testicular tissue [17, 18]. In order to infect and damage testicular cells, another necessary postulation is the arrival of the virus to the blood-testis barrier and its passing. While the latter can be achieved through cytokine-induced inflammation, which may alter the tight junctions of the Sertoli cells similarly to other viruses [19], the virus’s arrival to the testis requires the presence of viremia. Although nasopharyngeal swab samples are the reference specimens, SARS-CoV-2 has been reported in a wide range of body fluids and tissues, especially urine, feces, and blood [20]. While viral detection and loads may differ depending upon the sample characteristics, the severity of the disease and the timing from the primary infection, a SARS-CoV-2 viremia has been an inconstant finding [12, 20]. It should be further stressed that clinically evident orchitis in SARS-CoV-2 has been inconsistently reported [21–23], and recent reports and even a metanalysis showed an overall insignificant rate of detection of SARS-CoV-2 semen samples of COVID-19 patients [4, 12]. A relatively old study reported the chance of orchitis as a manifestation of SARS-CoV [24], and some recent studies from SARS-CoV-2 infections reported associations with testicular discomfort [25] or testicular pain [21]. The only certain association between testicular histological appearance of orchitis and presence of SARS-CoV-2 comes from post-mortem studies, where however the severity of the disease could have led to multiple organ failure, damage to the blood-testis barrier; as such, the orchitis-like appearance could rather be the result of a vasculitis secondary to COVID-19 related coagulation abnormalities of the testes vascularization [23]. In fact, the SARS-CoV-2-related testicular endothelial dysfunction may trigger inflammatory cells and leukocytes (CD3+, CD68+) in the interstitial tissue, which was found in some of these patients [26, 27]. Therefore, it is likely that the anatomical protection of the blood-testis barrier in association with the absent/low viremia in COVID-19 patients might hinder the SARS-CoV-2 progression towards the testes [28]. A recent paper showed results from an andrological follow-up of a cohort of SARS-CoV-2 recovered patients with no known previous andrological comorbidities, highlighting that after three months from recovery no persistent damage to testicular function could be observed [3]. In conclusion, it is relatively safe to assume that direct damage to the seminiferous tubules and spermatogenesis is a remote and probably clinically unimportant event in the vast majority of cases, at least in absence of significant comorbidities.

### Indirect damage to the testes: cytokines, inflammation, and fever

**Fig. 1:**
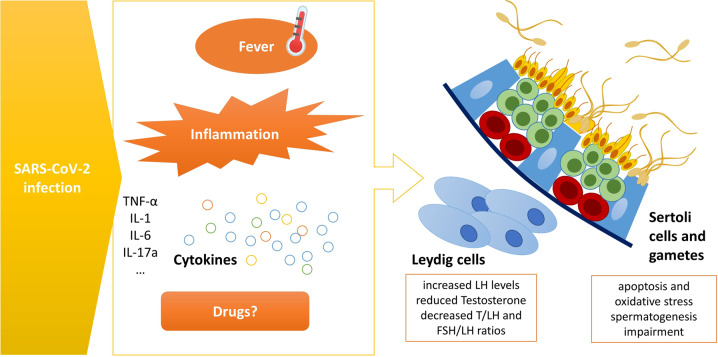
Indirect mechanisms through which SARS-CoV-2 disease could cause testicular damage and spermatogenesis impairment

It would be unwise, however, to lower the attention on male reproductive health after COVID-19, as testicular damage and spermatogenesis impairment may be caused by SARS-CoV-2 disease through different indirect mechanisms, mostly through the presence of inflammation, fever, and possibly gonadotoxic treatments (Fig. 1).

Fever may induce changes in testicular temperature that can also negatively impact germ cell development [29] and transiently affect semen quality and sperm DNA integrity [30]. As any scrotal temperature increase can exert detrimental effects on germ cell development [31], it is likely that fever induced by COVID-19 can alter sperm parameters even in the absence of the virus in the semen [32]. Several studies evaluated the effect of SARS-CoV 2 on semen quality showing impaired semen parameters. However, studies that evaluated subjects after a short time from recovery (median less than 30–40 days) are likely to show the effects of fever and treatments on semen analyses rather than a direct effect of the virus itself [33–36]. A few studies have considered semen analyses at least three months after recovery, that is, a complete “fever-free” spermatogenic cycle [37–42]. Metanalysis of these studies indicated that COVID-19 could result in short-term impaired sperm production [4], likely to the transient effect of fever and drugs. However, recovering patients should be monitored to determine whether andrological abnormalities are transient or persisting.

Testicular inflammation secondary to infections of the male reproductive tract is frequently associated with infertility [43]. Since COVID-19 is known to critically alter the concentrations of several pro-inflammatory mediators, up to what has been called “the cytokine storm” in the most critical cases, it is possible to imagine a role of these mediators in inducing testicular damage [44]. In fact, cytokines are a key element in defining the degree of background inflammation in the male genital tract, and their dysregulation secondary to infections may represent a risk factor for infertility [45]. Pro-inflammatory cytokines may alter the seminiferous tubule microenvironment significantly: many have been associated with alterations of tight junctions on Sertoli cells (Tumor Necrosis Factor α (TNF-α), interleukin-1 (IL-1), interleukin-6 (IL-6), interleukin-17a (IL17a) in particular), impacting the blood-testis barrier permeability and inducing oxidative stress and germ cell apoptosis [46–50]. IL-6 might also impact Leydig cell function resulting in the increased LH levels and decreased T/LH and FSH/LH ratios found in COVID-19 patients [39]. It is also possible that dysregulated cytokines and chemokines trigger an autoimmune reaction with consequences on testicular tissue [51], which may affect both semen quality (sperm concentration, motility) and the fertilizing ability of spermatozoa and the fusion of the gametes [52, 53]. Several papers have investigated the levels of cytokines (both pro and anti-inflammatory) in COVID-19 recovering subjects, which have been shown to be deregulated, often coupled with the concomitant alterations of markers of apoptosis and oxidative stress [35, 54]. SARS-CoV-2-related impairment of the gonadal hormone function with low Testosterone levels has also been observed [55–57]. Collectively, it could be hypothesized that an inflammation-related cytokine/chemokine dysregulation might induce Leydig and Sertoli cell dysfunction, thereby altering the gonadal hormonal axis and impairing the seminal antioxidant defense system, ultimately resulting in spermatogenesis impairment. Hopefully, these alterations will be transient, but long-term monitoring of recovering men at reproductive ages is highly recommended.

#### COVID-19 drugs and andrological health

Finally, the last and perhaps most underestimated aspect in post-Covid subjects is the iatrogenic contribution of certain drugs that may adversely affect the male reproductive health (Table 1). It is well known that many drugs are capable of interfering with spermatogenesis or are directly gonadotoxic [58]. The sudden onset of the SARS-CoV-2 has forced clinicians to use a number of hypothetically active drugs to manage the most severe cases, and recommendations for utilization of drugs have changed several times, pursuing the availability of new evidence. That was especially the case of hydroxychloroquine, an antimalarial drug whose indications also included rheumatological diseases such as lupus and rheumatoid arthritis. Since there was evidence of the chloroquine’s ability to interfere with SARS-CoV binding to the ACE2 receptor through inhibition of its glycosylation [59, 60], in the early months of the pandemic its use increased dramatically. Remarkably, after a few months of worldwide utilization, clear evidence of prolongation of the QTc interval and negative data on its use was published, leading to progressive discontinuation of its prescription in many leading centers [61–63]. However, animal data revealed that hydroxychloroquine treatment decreased sperm count and the weight of the testes and accessory sex organs, in association with a reduced testosterone secretion [64]. In vivo evidence is limited, but a relatively recent paper showed a clear association between the use of hydroxychloroquine and alteration of sperm parameters and increased sperm DNA fragmentation in patients with Systemic Lupus Erythematosus [65]. These alterations may also be secondary to imbalances of the pituitary-gonadal axis due to systemic inflammation. However, since these theoretical adverse effects have not been fully elucidated due to scant evidence, no specific recommendation can be made for patients who took this drug [58, 66]. It is, thus, advisable to maintain a high level of attention in the andrological follow-up of treated subjects.

**Table 1:** Summary of known effects of SARS-CoV-2 drugs on male reproductive health

Drug	Known effect on male reproduction	References
Hydroxychloroquine	Animal evidence of decreased sperm count, testes and accessory sex organs weight, and reduced testosterone secretion	[59–66]
	In vivo alteration of sperm parameters and increased SDF in patients with Systemic Lupus Erythematosus	
Antiviral drugs *(lopinavir/ritonavir, darunavir/ritonavir, molpunavir, nirmatrelvir-ritonavir, and remdesivir)*	*Lopinavir/ritonavir:* impaired sperm parameters (increased oxidative stress)	[67–73]
	*Darunavir/ritonavir:* minimal adverse effects on semen parameters in vivo	
	*Remdesivir:* insufficient data available on male fertility	
	*Molnupiravir:* known mutagenicity in vitro, if fatherhood is desired, at least 3 months discontinuation is recommended	
IL-6 receptor antagonist (tocilizumab)	Although not clearly teratogenic, there is insufficient data to make recommendations other than a careful follow-up of andrological health, as well as of the ensuing pregnancies	[73–81]
Corticosteroids	Hypercortisolism is known to disrupt pituitary-gonadal axis and fertility	[82–85, 88, 89]
	Hypercortisolism in animal models has been associated with reduced serum testosterone, Sertoli cell dysfunction, and impaired spermatogenesis	
	In vivo, hypercortisolism and steroid overtreatment have increased risk of infertility, hypogonadism and poor semen quality	
SARS-CoV-2 vaccination	No effects on semen parameters have been described	[91–94]

It should be reminded that most evidence is derived from the use of these drugs in other pathologies

A wide spectrum of antiviral drugs has been used in COVID-19 management, including lopinavir/ritonavir, darunavir/ritonavir, molpunavir, nirmatrelvir-ritonavir, and remdesevir. In general, antiviral and antiretroviral drugs are associated with alterations of semen parameters, although the available evidence is largely of low quality and often controversial since the confounding factor of the systemic viral disease is often difficult to ascertain [58, 67]. Animal experiments have shown that lopinavir/ritonavir have deleterious effects on sperm parameters, likely mediated by increased oxidative stress [68]. On the other hand, darunavir/ritonavir have been shown to have minimal adverse effects on semen parameters in vivo [69, 70]. Another drug, remdesivir, has minimal data available for male fertility but is used with no relevant adverse effects in pregnant women [71]. Molnupiravir, on the other hand, an antiviral agent targeting viral RNA‐dependent RNA polymerase, is known to be mutagenic in vitro. Although with less efficiency than viral RNA, molnupiravir can adversely affect the host DNA [72]. If fatherhood is desired, treated men should be warned of the potential genotoxic effect on sperm cell production, which is expected to endure for at least 3 months after molnupiravir discontinuation [73]. Although no evidence is currently available, the risk of molnupiravir’s genotoxicity in young male adults of reproductive age should be assessed with a careful andrological follow-up.

Tocilizumab (TCZ), an IL-6 receptor antagonist, has been recommended in association with corticosteroids in severely critical cases of COVID-19 [73]. IL-6 signaling, together with other cytokines and chemokines, plays a role in testicular function [74, 75], and it is likewise known that its dysregulation is present in several andrological diseases [76–79]. High testicular levels of IL-6 were associated in vitro with disruption of the blood-testis barrier integrity due to alterations of tight junction proteins expression, and apoptosis of germ cells, through increased oxidative stress [79]. Analysis of testicular tissues from autopsies has confirmed that IL-6 levels are increased in SARS-CoV-2 infected subjects, in association with a dysregulated expression of junctional proteins (occludin, claudin-11, connexin-43), decreased numbers of Sertoli cells, and decreased sperm counts [80]. Therefore, blockading the IL-6 signaling might be beneficial for COVID-19 subjects at multiple levels on the theoretical level, though no specific data is available on this topic. Although not clearly teratogenic, data from the use of Tocilizumab in reproduction and pregnancy is still insufficient to make recommendations [81] other than a careful follow-up of andrological health, as well as of the ensuing pregnancies.

Various corticosteroid formulations are used to manage severe to critical cases of COVID-19. The role of hypercortisolism in disrupting the pituitary-gonadal axis and fertility is well known [82]. Animal models indicate that induced hypercortisolism is associated with reduced serum testosterone, Sertoli cell dysfunction, and impaired spermatogenesis [83–85]. Nonetheless, glucocorticoid administration may benefit spermatogenesis in certain pathological conditions [86]. In fact, Sertoli and Leydig cells express the glucocorticoid receptor, and its signaling within physiological levels is necessary for their maturation and function [87]. In vivo evidence suggests that adolescents with hypercortisolism have an increased risk of infertility [88], and men with congenital adrenal hyperplasia overtreated with corticosteroids may present hypogonadism and poor semen quality [89]. Available knowledge of corticosteroid impact on spermatogenesis is mainly known from subjects undergoing chronic treatments, while COVID-19 survivors are likely to have taken short-term high-dose corticosteroid treatment. In general, however, it cannot be excluded that even a short-term corticosteroid treatment affects semen quality at sufficiently high doses. Although the balance between the benefits and risks clearly moves towards the first, we should carefully evaluate men who performed glucocorticoid treatments after COVID-19, especially in the presence of preexistent andrological diseases.

### Vaccination

Another troubling question that arose during the pandemic was whether the available vaccines, the best weapons against SARS-CoV-2, could have possibly recoiled back by impairing the male reproductive potential. An internet-based study demonstrated that, after approval of vaccinations by health authorities, queries on fertility effects of SARS-CoV-2 vaccination increased dramatically [90], reflecting fears of many young adults of reproductive age, despite the lack of a solid theoretical link. However, the few available papers concur that no effects on semen parameters, oxidative stress and seminal plasma IL-6 are present [91–94]. A recent meta-analysis of these studies reported that in healthy volunteers receiving mRNA-based vaccines, no significant negative effect on sperm concentration motility and semen volume was observed, while data on sperm morphology was unavailable [4].

## Conclusions

A careful andrological evaluation of men recovering from COVID-19 is highly recommended. Although the likelihood of direct testicular damage from SARS-CoV-2 is somewhat low, there are many indirect ways through which the andrological health may be disrupted, and the role of coexisting pathologies and comorbidities cannot be excluded. Fever, cytokine imbalance, and drugs are capable of transiently altering the pituitary-gonadal axis and spermatogenesis. In particular, many drugs have been used to treat the various COVID-19 manifestations, and, likely, new evidence will show up, further modifying the drugs selection and indications. Since evidence is relatively scarce, a careful andrological follow-up and counseling of these patients are mandatory. On the other hand, available evidence shows high safety regarding andrological health for available vaccines, although evidence is mainly restricted to mRNA vaccines.
